# The Eurofever Registry for autoinflammatory diseases: results of the first 15 months of enrolment

**DOI:** 10.1186/1546-0096-9-S1-P303

**Published:** 2011-09-14

**Authors:** N Toplak, J Frenkel, S Ozen, F De Benedetti, M Hofer, I Kone-Paut, H Girschick, B Neven, H Ozdogan, J Kummerle-Deschner, J Arostegui, A Simon, S Stojanov, R Vesely, C Wouters, V Hentgen, C Rose, P Dolezalova, H Lachmann, P Woo, I Touitou, A Martini, N Ruperto, M Gattorno

**Affiliations:** 1 IRCCS G. Gaslini, Pediatria II, Paediatric Rheumatology International Trials Organisation (PRINTO

## Background

The main limitation to a better knowledge of Autoinflammatory diseases is related to the fragmentation of the diagnosed cases in different centers.

## Aim

To evaluate the number of patients enrolled in the Eurofever Registry in the first 15 months of enrolment.

## Methods

A web-based registry collecting baseline and clinical information on Autoinflammatory diseases is available on the PRINTO web-site (www.printo.it). The registry is open to all Pediatric Rheumatology centers and adult centers with a specific interest in Autoinflammatory diseases. The following monogenic autoinflammatory diseases were considered: FMF, CAPS, TRAPS, MKD, Blau’s syndrome, PAPA, DIRA, NLRP12-mediated periodic fever. Information on CRMO, Behcet’s disease, PFAPA and undefined periodic fevers were also collected.

## Results

During the first `15 months 1360 (M:F=652:708) patient data from 64 centers in 28 countries have been entered in the registry. So far clinical and genetic information on the following patients has been collected: 376 FMF pts; 156 TRAPS pts, 128 CAPS pts, 87 MKD pts; 13 Blau’s syndrome pts; 7 PAPA pts, 3 pts with NLRP-12 mediated periodic fever and 2 DIRA pts. In total 17 new mutations have been reported. Data on 257 PFAPA patients, 135 CRMO, 55 Behcet’s disease and 141 patients with undefined periodic fever are also available.

## Conclusions

A common registry for collection of patients with Autoinflammatory disease is available and the enrolment is ongoing. Data will be available for analysis of the clinical presentation, outcome and response to treatment of Autoinflammatory diseases and for comparative studies among different Autoinflammatory conditions.

